# An Endless Quest

**Published:** 1890-09-27

**Authors:** 


### The Hospital

A Weekly Journal of

**Science, Medicine, Nursing, and Philanthropy.**

*For Hospitals, Asylums, Medical Practitioners, Students, Nurses, Families, Parishes, Congregations, and the Charitable Public.*

Vol. VIII.—No. 209.] [September 27, 1890.

### An Endless Quest.

"What is the use of your scientific medicine, since it leaves the patient very much where he was before? The consumptive was not cured a thousand years ago, and he is not cured to-day; the victim of cancer is doomed to as hopeless a future as she was in the days of Miriam, the sister of Moses, and other Hebrew women skilled in the use of herbs. You patch here and you tinker there; but the real enemies that lie in the pathway of mankind between the cradle and the grave you neither scotch nor kill. Your promises are profuse, aud your learning prodigious; but where is the solid fruit of your labours?"

Such are the questions addressed to scientific medicine by some of the practical and progressive intellects of the time. Satisfying answers are seldom forthcoming; and so the generations of such men pass on their way, casting a contemptuous look at medicine, and wholly sceptical of its scientific and practical value. Men of the same intellectual mould have impatient doubts about the truth and worth of religious faith. They see the average teacher of religion clothed in his peculiar garments; they hear him spin his threadbare platitudes in the pulpit; they know his ready formulæ, by which he thinks, or tries to think, that every fact of science and every agony of man may be explained; and they do not deeply trust either him or his teachings. They are quick to discern, too, the absence of results. The lustful man is not cured of his lusts by the priest, any more than the consumptive is cured of his consumption by the doctor; the weak and wicked fool remains weak and wicked in spite of theological and ecclesiastical nostrums, exactly as the hypochondriac keeps his tendency to hypochondriasis, or the scrofulous man his debility, notwithstanding all the cholagogues and tonics which the physician can invent, or the laboratory of the pharmacist bring forth. Religion, which is as old as the world, fails either to cure sin or to banish misery. Medicine, which is as old as religion, neither uproots disease nor annihilates death. What is the practical use of the one or the other; and why should men any longer submit to the incubus of either?

Necessity is a stern task-mistress! The "constitution and course of Nature" may be studied, but where is the scientific Hercules who is equal to the task of altering it? Is not that the explanation of what we call sin, and of what we know to be misery? And is it not also the explanation of disease and death; and of the doctor's powerlessness to do more than ripple the surface of the stream of human life? The strong, bold, and inexperienced intellect is fixed in its resolution to do something which shall be absolute, final, complete. The vicious man shall be regenerated, and become a perfect saint: an imperfect saint is not worth the making. The feeble consumptive shall have his disease destroyed and uprooted, and he himself shall be made a model of health, vigour, and happiness: to half cure such a man, and to leave him feeble, pale, and inefficient, is worse than doing nothing at all. So reasons the powerful and resolute intellect of the inexperienced man. Nature sometimes permits us to reach such finished and perfect results, but not often.

It is not well that a man should lower his aims as life advances and experience opens his eyes. It is a calamity rather, and a sign of weakness. Nor ought he ever to be content with results that fall short of the best. But he has to find out that though he always aims at the highest he is frequently compelled to accept second or third rate results as the only possible ones. He learns in time to put all his strength into work which may lead to a comparatively insignificant end, and to be thankful for a small good; thankful, though not satisfied.

Is it of any use to wear out brain and heart in the pursuit of a medical science which yields such poor fruits? It is a futile question. The sick man will importune the physician to the end of time, and the physician can no more help responding to the appeal than the air can refuse to vibrate into music at the stroke of the cathedral bell. What is the use of your philosophy, contemptuously asks the "practical" man? For answer, the philosopher goes on philosophising, and he tells the practical man that he, too, philosophises, though he does not admit the use of it. The sceptic is astonished at the religions of the world; to him they are madnesses. But the world continues to be as religious and as "mad" as before. Nature has so designed it; and we may just as well try to gash the sky with a sword as to alter the constitution of the world. Therefore—and the logic is as complete in fact as in form—therefore, there must be an art of medicine, because the world has a habit of sickness and disease. And since there must be an art, it is imperative that there should be a science, if only in the interests of public morality, for art divorced from science tends rapidly to moral corruption. And though the physician cannot always cure and make a finished work, as the engineer can who builds and completes his bridge, yet he will steadily keep up the quest for new knowledge, whilst he puts to the best daily use such knowledge and skill as he has already acquired.

That is rather a dreary view of the medicine of the latter half of the nineteenth century. Dreary? Yes; and not altogether square with fact. Medicine is not a failure any more than religion or navigation is a failure. All the people in the world are not saints by any means; but the general manners of civilised man are much gentler, much juster, much more reasonable than they were, even so recently as a thousand years ago. We have compared medicine to navigation, as well as to religion. The "practical" man thinks we have given ourselves away in so doing. By no means! The art of navigation is revolutionised by steam; man's victory over the ocean is apparently complete, since he can make headway against the fiercest opposing winds, and ride buoyantly on the mountain summits of the loftiest and most resistless waves. The victory, however, only appears to be complete. Are collisions at sea unknown? Does no steamship ever founder and go down with all hands? Is a "ship on fire" a tragedy of the past? Are there no sailors' widows and orphans in the land? It is true that our mastery of the waters is wonderful and glorious compared with that of the Greek navigators or the Romans who held the world in thrall. But the sea is still a terror, a dread, a mystery, a conqueror, which we can neither subdue nor move from its place.

We admit with just pride the prowess of man in so far rising superior to the might of the ocean that he can make use of it in all its moods. That our forefathers could not do. But as striking as is the victory of the modern navigator over the great waters, so striking is the victory of the modern physician over many forms of disease and agents of death. Is it nothing to have banished superstition from medical practice, and to have established reason and experiment in its stead? Is it nothing to have put a bridle into the jaws of plague and pestilence? Is it nothing to have carried surgery into every cavity of the human body and to have wrung triumph out of apparently hopeless defeat? Medicine has not done everything! No; but it has done much. Medicine will never banish disease and annihilate death! No; but it will in no long time make men so familiar with all the causes of sickness and disease that they will resolutely and intelligently remove them; and so doing, it will reduce the agents of destruction to a minimum both in number and destructiveness; and it will thus have only the milder forms of disease left to cope with, and will fight them with ever increasing confidence and success. Scientific medicine has, during the last few decades, done much; but it is only at the very beginning of its triumphant march. The time is undoubtedly coming when every man will "know himself" as a physical organism, and will know the dangers of the environment in which he lives. When that time shall be fully here, it will be recognized that the religion of the body is one with the religion of the soul; and that both these religions, intelligently understood and honestly carried into practice, have in them the seeds and elements of a perfect life.

